# NanoZnO-modified titanium implants for enhanced anti-bacterial activity, osteogenesis and corrosion resistance

**DOI:** 10.1186/s12951-021-01099-6

**Published:** 2021-10-30

**Authors:** Zheng Wang, Xiaojing Wang, Yingruo Wang, Yanli Zhu, Xinqiang Liu, Qihui Zhou

**Affiliations:** 1 Institute for Translational Medicine, Department of Orthodontics, The Affiliated Hospital of Qingdao University, Qingdao University, Qingdao, 266003 China; 2 School of Stomatology, Qingdao University, Qingdao, 266003 China; 3 Department of Oral Implantology, The Affiliated Hospital of Qingdao University, Qingdao, 266003 China; 4 Shandong University of Science and Technology, Qingdao, 266590 China

**Keywords:** Titanium implants, Nano-ZnO, Anti-bacteria, Osteogenesis, Anti-corrosion

## Abstract

Titanium (Ti) implants are widely used in dentistry and orthopedics owing to their excellent corrosion resistance, biocompatibility, and mechanical properties, which have gained increasing attention from the viewpoints of fundamental research and practical applications. Also, numerous studies have been carried out to fine-tune the micro/nanostructures of Ti and/or incorporate chemical elements to improve overall implant performance. Zinc oxide nanoparticles (nano-ZnO) are well-known for their good antibacterial properties and low cytotoxicity along with their ability to synergize with a variety of substances, which have received increasingly widespread attention as biomodification materials for implants. In this review, we summarize recent research progress on nano-ZnO modified Ti-implants. Their preparation methods of nano-ZnO modified Ti-implants are introduced, followed by a further presentation of the antibacterial, osteogenic, and anti-corrosion properties of these implants. Finally, challenges and future opportunities for nano-ZnO modified Ti-implants are proposed.

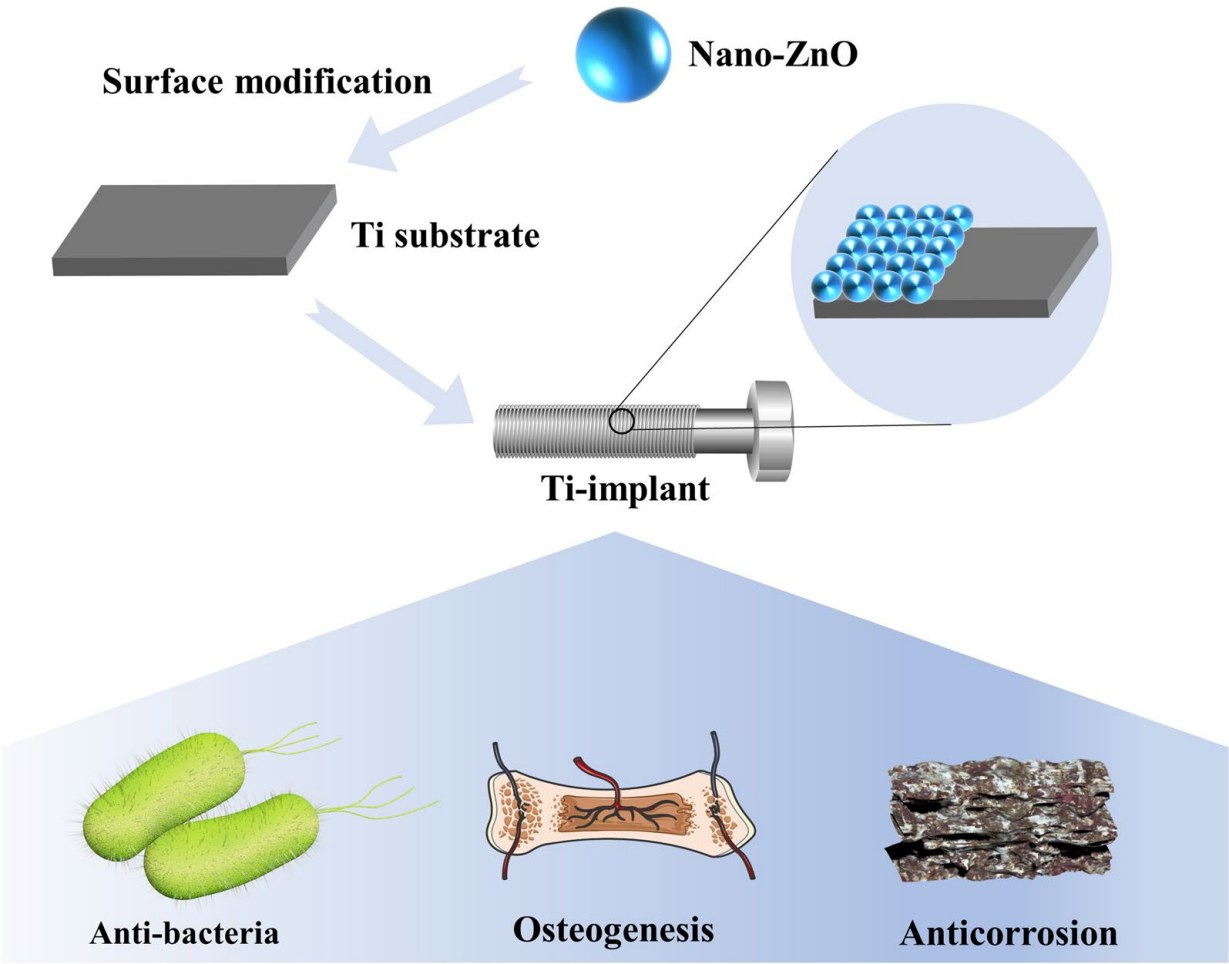

## Introduction

The development of implants with both high strength and biosafety has long been a hotspot in the field of biomedical engineering [1–3]. Due to their excellent corrosion resistance, superior mechanical properties and biocompatibility, titanium (Ti) implants have been widely used in orthopedic surgery, oral implantation, and other medical devices [4–6]. However, two major issues have arisen in their clinical application: implant-related infection and poor osteogenesis [7–10].

The microbial attachment associated with Ti-implants is the major cause of postoperative implant infection. To date, microbial infection remains one of the most common and serious clinical complications after traumatic implantation [11]. Microbial infection is commonly caused by bacterial adhesion and biofilm formation on the implant surface [12]. In the clinic, implant-associated bacterial infection can lead to implant loosening in situ and soft tissue damage. Such tissue damage often leads to chronic and/or recurrent diseases if not treated timely and properly [13, 14]. Besides, bacteria bonded to the surface of the implant to form a biofilm that prevents cells from attaching to the implant surface, further aggravating the loosening of the implant in bone tissue [12]. In patients with poor bone quality, the repair of necrotic tissue is difficult to manage due to the poor vascularity of infected bone tissue, resulting in a particularly long treatment process with a poor prognosis [15, 16].

Another tough problem with Ti implants that remains to be solved is their insufficient osteogenic, osteoinductive, and osseointegration abilities [17, 18]. Commercial Ti-implants cannot completely meet the demand of clinical applications due to their bio-inert nature, and they are easily covered by a dense oxide film, particularly under conditions of poor or insufficient bone. Besides, Ti-implants are mainly bonded in the body through physical chimerism, which leads to a lack of stability and a tendency toward loosening or shedding with long-term use [19]. Only through excellent osseointegration can an implant be guaranteed to be stable and safe in the long term. In addition, there is a difference in the coefficient of thermal expansion between Ti-implants and bone, which also contributes to the unstable bond between them. Therefore, how to effectively improve the osseointegration of implants has long been of great concern. Ideal osseointegration shown under light microscopy is characterized by close apposition of the implant to the adjacent bone tissue without any fibrous tissue infiltration [20–23]. Mechanical treatment of Ti implant surfaces has been demonstrated to effectively improve the mechanical clamping force between the implant and the bone tissue. However, in clinical applications, the actual bonding ability is still not sufficiently sustainable [24, 25]. Furthermore, the implants bond slowly to bone tissue, usually over 3–6 months, and the prolonged recovery time resulting from this slow bonding also limits the application of Ti implants. Indeed, improving the osseointegration of Ti implants requires a better understanding of the mechanisms of induced osteogenesis. In addition, researchers have recently realized that the immune system may play a role in bone regeneration that should not be underestimated. Immune cells, such as macrophages and T cells, actively balance osteoclastogenesis and osteogenesis by secreting molecules such as RANKL protein, interferon-gamma, and inflammatory cytokines in communication with various bone cells [26, 27]. Although biomaterials for bone regeneration have been studied progressively over the years, the role played by immune cells in this process has not been fully demonstrated [28]. Therefore, a deeper knowledge of the role of immune cells in the integration of biomaterials for bone regeneration may help overcome the current dilemma of inefficient osseointegration of implants.

It is also worth noting that during the post-implantation period, Ti implants are exposed to the electrolyte environment of human body fluids, which undergo biochemical reactions at the interface between the Ti implant and bone tissue [29]. These adverse reactions in the physiological environment could lead to electrochemical corrosion of the implant surface, thereby reducing the longevity of the Ti implant. To this end, surface treatment of Ti implants is an effective way to prevent postoperative implant infection, improve the ability of osseointegration, and enhance corrosion resistance. Therefore, it is generally considered that surface modification is a direct and effective approach to endow new interfacial properties to Ti implants while retaining their original advantages [30].

In recent decades, nanomaterials have been found to possess an ultra-small size, high specific surface area, high reactivity, tunable surface modification capacity, and antimicrobial activity [12, 21, 31–35]. Meanwhile, the application of nanotechnology in biomedicine is rapidly becoming the main driving force behind the changes that are taking place in the field of antimicrobials as well as tissue repair and regeneration [36–42]. In particular, inorganic nanoparticles, such as ZnO, CuO, and Ag nanoparticles, have shown effective antimicrobial activity against pathogenic microorganisms and have been widely used on implants [43, 44]. However, large amounts of heavy metals in implants (e.g., Ag and Cu) accumulate in human bones and other tissues over time, with serious chronic toxicity effects [45].

Notably, as an amphoteric metal oxide, ZnO has attracted wide attention for its strong and broad-spectrum antimicrobial, antitumor, low toxicity, and various other properties [46–48]. In particular, nanoscale ZnO particles (nano-ZnO), which have been approved for human use by the U.S. Food and Drug Administration (USFDA), exhibit attractive antibacterial properties because Zn^2+^ and reactive oxygen species (ROS) are released by nano-ZnO [49, 50]. Also, zinc is an essential trace element in cell development, DNA synthesis, enzyme activity, and biomineralization [51]. A proper amount of zinc on the surface of biomaterials has been confirmed to stimulate bone cells and induce a range of in vivo behaviors, e.g., adhesion, spreading, proliferation, osteogenic differentiation, osteogenesis, and mineralization [48, 52, 53].

**Fig. 1:**
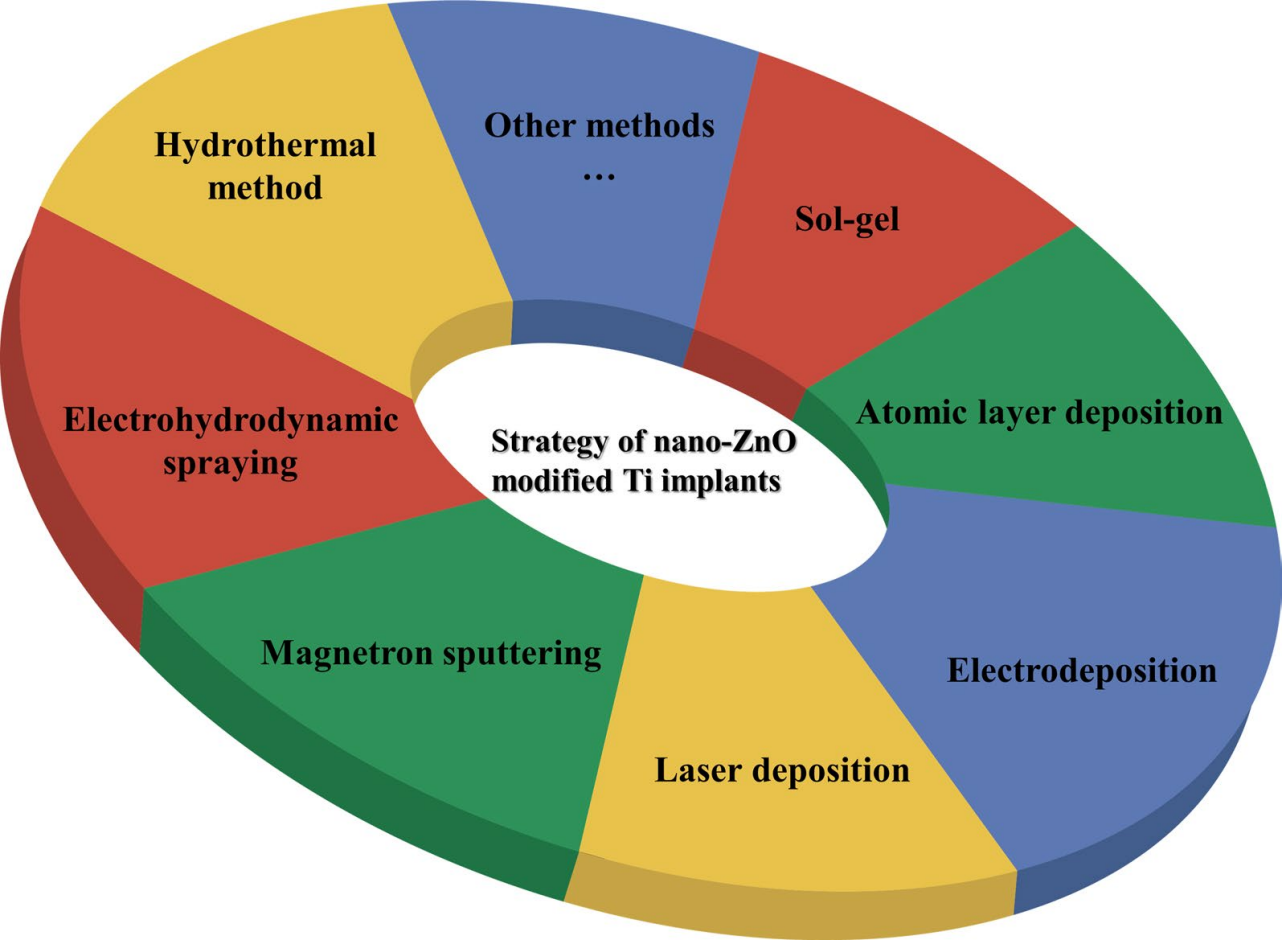
Main strategies of nano-ZnO modified Ti implants

In this review, current strategies in the use of nano-ZnO material modified onto the surface of Ti implants were summarized (Fig. 1) and the relevant literatures involved were also categorized (Table 1). Next, we highlighted the enhancement properties of nano-ZnO coatings on Ti implants in terms of antimicrobial, osteogenesis, and anti-corrosion abilities. Finally, future challenges and opportunities in the clinical use of nano-ZnO coatings on Ti implants were discussed.

## Fabrication of nano-ZnO modified Ti materials and their biosafe properties

### Methods for preparing nano-ZnO modified Ti implants

#### Electrodeposition

Electrochemical deposition is a technique in which an electric current is passed through an electrolyte solution under the action of an external electric field and a redox reaction occurs at the electrode to form a coating. As an in situ formation technique, electrochemical deposition can form well-adhered, uniformly distributed, and high purity nano-ZnO at various Ti-substrate interfaces, which is particularly suitable for surface modification of biomedical implants. Studies have found that both the zinc content and the fine-tuning of the hierarchical topography can be controlled by adjusting the timing of the electrochemical deposition of ZnO nanorods. ZnO nanorods were electrodeposited on the walls of pores prepared by micro-arc oxidation (MAO) to produce a micro/nano hierarchical structure suitable for biomedical applications [54]. It was found that the density of ZnO nanorods on the surface of the Ti-substrate changed with the duration of deposition, and the longer the deposition time was, the higher the density [55]. When the concentration of Zn^2+^ ions in the electrolyte was 2.5 mM, the diameter of the ZnO nanorods was approximately 200 nm, and the length was approximately 2 μm. However, when the Zn^2+^ ion concentration was increased to 5 mM, a larger ZnO nanorod diameter was observed, reaching 300 nm with a similar length of approximately 2 μm. The above results show that modulating the concentration of Zn^2+^ ions during electrodeposition has little effect on the length of ZnO nanorods, although it is capable of causing a drastic change in diameter [55]. Similar results were observed by Yao et al., in which irregular ZnO nanorods were doped into TiO_2_ nanotubes (TNTs) in the electrodeposition method by varying the concentration of the electrolyte bath solution and reaction time [56]. In addition, Chang et al. used an electroplating method to deposit ZnO coatings with different thicknesses onto Ti plates at voltages of 2.0, 2.25, and 2.5 V. Bacterial adhesion was lowest in the 2.5 V ZnO sample, indicating the highest antibacterial activity [57]. Moreover, a ZnO-containing TiO_2_ coating can also be prepared in one step by employing the MAO technique using electrolytes containing Zn^2+^ ions, and this nano-ZnO modification strategy is more efficient [51, 58].

Apart from single ZnO coatings, composite coating fabrication is another approach to improve antibacterial properties [59]. Similarly, hybrid coatings prepared by electrodeposition of functional substances [e.g. Sr^2+^ ions and hydroxyapatite (HA)] doped in nano-ZnO electrolytes have been used to enhance the corrosion resistance and biocompatibility of Ti implants [60]. However, when direct current is applied, there is a tendency for concentration polarization and the formation of loose and poor adhesion coatings on the substrate surface. Recent studies have shown that adopting pulsed electrochemical deposition is an effective way of controlling the reaction rate and has the ability to trigger intermittent energization mechanisms during operation [61], which reduces the electrolyte concentration polarization, increases deposition efficiency and generates nano-ZnO crystals of uniform size on the surface of Ti substrates.

Electrophoretic deposition (EPD) is also an attractive method that uses the phenomenon of charged particles moving in an electric field to apply coatings of different thicknesses onto a Ti implant surface and is suitable for large and complex shapes of implants [62]. One study used different deposition voltages to electrophoretically deposit coatings onto a Ti-implant surface for the same time and then sintered them in an air/vacuum atmosphere. The results showed that the hardness values of the samples and the denseness of the coatings are related to the gas environment and the voltage intensity. [62]. Electricity plays an important role in electrodeposition technology, but accurate preparation of the required morphology and quantity of nano-ZnO on the surface of Ti substrates remains to be solved in the future.

#### Atomic layer deposition

Unlike electrodeposition, atomic layer deposition (ALD) can accurately control the thickness of the deposited film at the atomic scale or monolayer level, which facilitates the preparation of homogeneous coating on the surfaces with specific conditions (e.g. complex micro/nano structures, high aspect ratios, porous surfaces) [63, 64]. The 1D nanostructure of ZnO on carbon nanotube/chitosan-modified Ti implants produced using atomic layer deposition was first introduced by Zhu et al. By adjusting the cycling number, the thickness of ZnO and the Zn content can be precisely controlled, which not only regulated the proliferation and osteogenic differentiation of osteoblasts but also showed good antibacterial properties [52]. In the same way, the ZnO seed layer was also prepared by atomic layer deposition (ALD), which has uniform and narrow particles with an average size of 20 nm, and the thickness of this layer was approximately 40 nm [65]. However, as the cycling number gradually increases from 0 to 50, the biocompatibility of the material shows a tendency to ramp up and down, indicating the toxicity of nano-ZnO sheets with excessive thickness [48]. Therefore, the control of cycle parameters in ALD technology is a decisive means to affect the biological properties of materials.

#### Magnetron sputtering

The magnetron sputtering method is widely used to produce nano-ZnO coatings, in which the apparatus mainly used a highly pure ZnO as the target and medical Ti metal plates as the substrate for depositing nano-ZnO. By precisely regulating the deposition time during the sputtering process, ZnO specimens ranging from discrete nanoparticles to continuous films were prepared, which led to a significant development in the surface morphologies [66]. On the surface of Ti6Al4V, Ding et al. successfully prepared a ZnO-doped tantalum oxide multilayer composite coating (ZnO-Ta_x_O_y_) by magnetron sputtering which showed that the ZnO-Ta_x_O_y_ coating has great potential in improving the corrosion resistance and antimicrobial performance of Ti6Al4V implants. However, the cytocompatibility and optimal design of ZnO-Ta_x_O_y_ coatings (e.g., composition, coating thickness, preparation parameters) need to be further improved [67].

#### Laser deposition

As is well-known, it is difficult to simultaneously immobilize several components on the surface of metallic implants. Laser cladding has a broad application prospect in the modification of implant surfaces, which can change the specific surface area, surface roughness, and wettability of implants. Studies have reported that the composite coatings composed of Ag nanoparticles (nano-Ag), nano-ZnO, and HA nanopowders with different ratios were prepared on the Ti6Al4V alloy by laser cladding, which provided good adhesion between the coatings and substrate because of local melting during the laser treatment [46]. Comparatively, laser ablation in a liquid environment is a potential alternative to surface modification of Ti substrates as it has excellent characteristics of eliminating sharp surface features and narrowing down the size distribution of nanoparticles [68]. Zhao et al. successfully developed ZnO-coated microgrooves on Ti-6Al-4 V implants using a two-step nanosecond laser processing technique, which achieved a substrate surface with target morphology as well as rapid fixation of chemical composition [69]. However, this technique should be avoided in conjunction with the organic component of the biomodified Ti surface, as the high local temperature generated by the laser could be a serious cause of loss of biological activity.

#### Electrohydrodynamic spraying (EHDA)

In contrast to electrochemical deposition, the EHDA method deposits nanoparticles on the surface of the substrate by loading a suspension containing nanoparticles into a spray device and energizing it to deliver a fine spray. One study used a stainless steel needle with a diameter of 300 μm to spray the content of a 1 mL syringe onto the substrates, delivering an optimized coating through a symmetrical conical jet spray to ensure coverage of the sample [70]. Electrostatic spraying is a simple and economical way to prepare nano-ZnO coatings. Particularly, it is advantageous for coating materials while maintaining their biochemical properties at low temperatures.

#### Sol-gel

Sol-gel technology, by which composite organic-inorganic materials are prepared at low temperature, involves hydrolysis of the constituent molecular precursors and subsequent polycondensation into a glass-like form. It is used for its ability to mass-produce nano-ZnO films at low cost and with favorable substrate adhesion. For instance, Trino et al. used a modified sol-gel Pechini method to obtain the ZnO resin precursor. The Ti surface was then treated with a strong oxidant, Piranha solution, to form hydroxyl groups, which are extremely hydrophilic and able to bond with ZnO to improve the adhesion of oxide thin film. The ZnO deposition (~100 nm) was performed by three sequential spin coatings on a Ti substrate [71]. Taking advantage of the fact that ZnO films have hydroxyl groups at the end of the surface, these hydroxyl groups can be functionalized by 3-(4-aminophenyl) propionic acid (APPA) and 3-mercaptopropionic acid (MPA) molecules, thus enabling effective loading of DMP1 peptides, resulting in a more active and aggressive surface. [72]. For a better antibacterial property, AgNO_3_ was integrated into the ZnO sol to obtain the ZnO/Ag sol [73]. Although the sol-gel method is simple and convenient, it is inevitable to mixed with other methods to prepare Ti-based nano-ZnO thin films.

#### Hydrothermal method

The hydrothermal method has been widely used in the preparation of ZnO with nanorod structures. Changing the factors, such as reaction temperature, vessel pressure, and reaction time, may affect the growth of ZnO nanocrystals on the substrate surface [74, 75]. Seed layers can be prepared on the surface of Ti substrates and then hydrothermally treated to aid their growth [76, 77], or nano-ZnO can be prepared directly on the substrate surface in a sealed pressure vessel [74]. The method of preparing nano-ZnO coatings by dissolving the Zn-containing compound in a heated solvent and crystallizing the nanoparticles on the substrate surface has the advantages of complete grain development, high purity and uniform distribution, and the possibility of using cheaper raw materials compared to other methods. However, although the hydrothermal method has been widely used in the study of preparing ZnO with nanorod structures, its characteristics, such as the time-consuming preparation process and the relatively single coating composition, should not be ignored.

#### Other methods

Ti substrates can also be coated with hybrid nanoparticles containing-ZnO by the drop dipping method through hydrogen bonding [78–80]. Similarly, spin-coating is also another simple method for preparing thin films, which is often used in combination with other methods and has been mentioned in the previous section of this article [71, 72, 81, 82]. In addition, a mix of applications is also assigned to this section [83].

### Biosafe properties of nano-ZnO modified Ti implants

The biosafety of modified Ti implants has always been a concern for a wide range of research groups. To ensure reliable implantation and long-lasting use in vivo, biocompatibility is an essential characteristic that the material must possess [4, 84, 85]. The cytotoxicity of nano-ZnO has been discussed, despite its relatively low toxicity, its application in bone still requires attention [86, 87]. The mechanism of toxicity of nano-ZnO was reported to be closely related to the excessive release of Zn^2+^ ions and ROS generation causing cell death [88]. Excess nano-ZnO could produce cytotoxicity and be detrimental to cellular growth, which has been confirmed in previous studies [52, 63]. Additionally, the bonding form of nano-ZnO to the Ti substrate also impacts its biocompatibility. In one study it was shown that ZnO nanorod-coatings fabricated by the hydrothermal method were more stable and reliable than nanosphere-coatings prepared by the drop dipping method, implying that an unstable modified interfacial structure would potentially result in the generation of large amounts of free nano-ZnO with excessive toxic effects in the local microenvironment [79]. But, when nano-ZnO is bound to the substrate in the same or a similar way, the cytotoxicity also varies according to its morphology, that is, rod-shaped structure inducing lower cell viability versus spherical structure [65]. The previous study has demonstrated this result and interpreted it as the lack of initial spreading on ZnO nanorods, which is not conducive to cell adhesion and leads to cell death [89]. Besides, modulating the releasing behavior of nanoparticles is also a key point to the biocompatibility of nano-ZnO modified Ti materials. The utilization of functional coatings [e.g., polydopamine (PDA), chitosan, and poly(lactic-co-glycolic acid) (PLGA)] enables effective regulation, which can mostly be attributed to physical blocking, ion chelation, and antioxidant effects [52, 65, 76, 90]. These results suggest that the nano-ZnO modification on Ti holds promise for clinical use, but the potential toxicity risks still remain to be minimized by innovative design.

**Table.1:** Overview of different fabrication strategyies, morphologies, and doping elements of nano-ZnO modified Ti and their applications

Strategies	Morphologies	Doping elements	Applications	References
Electrodeposition	Nanoparticles	Ag	Anti-bacteria	[54]
	Nanoparticles	–	Anti-bacteria, anti-inflammation inhibition	[56]
	Nanoparticles	–	Anti-bacteria, anti-corrosion	[58]
	Nanoparticles	HA, Ag, Cu	Anti-bacteria, osteogenesis	[61, 91]
	Nanorods	–	Not reported	[55]
	Nanorods	–	Anti-bacteria	[57]
	Nanospheres, nanoflakes, worm-like and flower-like structure	–	Anti-corrosion	[92]
	Not reported	Ag, chitosan, gelatin	Anti-bacteria	[59]
Electrophoretic deposition	Nanoparticles	–	Anti-corrosion, anti-bacteria	[62]
Atomic layer deposition	Nanoparticles	Sr, octadecylphosphonic acid-toluene	Anti-bacteria, osteogenesis	[64]
	Not reported	–	Anti-bacteria, osteogenesis	[48]
	Nanoparticles	Chitosan	Anti-bacteria, osteogenesis	[52]
Magnetron sputtering	Nanoparticles	–	Anti-biofilm, immunoregulation	[66]
	Not reported	Ta_x_O_y_	Anti-bacteria, anti-corrosion	[67]
Laser deposition	Not reported	Ag, HA	Anti-bacteria, osteogenesis	[46]
	Not reported	–	Osteogenesis	[69]
Electrohydrodynamic spraying	Nanoparticles	HA	Anti-biofilm	[70]
Sol-gel	Not reported	Ag	Anti-bacteria, anti-corrosion	[73]
Hydrothermal method	Nanorods	Ag, PLGA	Anti-bacteria, osteogenesis	[76]
	Nanorods	Ag	Anti-bacteria	[77]
	Nanoparticles	–	Anti-bacteria, osteogenesis, immunoregulation	[53]
	Nanoparticles	–	Anti-bacteria, osteogenesis	[74]
	Nanoparticles	HA	Anti-bacteria	[93]
Other methods	Not reported	Functional molecules	Osteogenesis, anti-corrosion	[71, 72]
	Quantum dots	Antibiotic, folic acid (FA)	Anti-bacteria, seal-platform	[81]
	Nanoparticles	Chitosan	Anti-bacteria, anti-corrosion	[80]
	Nanoparticles	N-halamine, polystyrene-acrylic acid, SiO_2_	Anti-bacteria, osteogenesis	[78]
	Not reported	HA	Anti-bacteria	[82]
	Nanorods-nanospheres hierarchical structure	–	Anti-bacteria	[79]
	Nanorods-nanoslices hierarchical structure	–	Anti-bacteria	[83]

## Antimicrobial activity of nano-ZnO modified Ti implants

### Antimicrobial and antibiofilm mechanisms of nano-ZnO

#### Generation of ROS

The generation of ROS is the most widely studied and accepted mechanism underlying the antibacterial activity of ZnO. ROS include highly reactive ionic species, and free radicals such as O^−·^, HO·_2_, H_2_O_2,_ and HO·. They can damage DNA, cell membranes, and cellular proteins, and can further lead to bacterial cell death by inducing an oxidative stress response [94]. Disturbance of the balance between generated ROS and their reducing equivalents is termed oxidative stress [95–98]. Bagchi et al. fabricated the ZnO-squaraine (SQ) dye nanohybrid aroused by near-infrared light. The time-resolved fluorescence transient experiments verified the photoinduced interfacial electron transfer process from SQ excited state to ZnO conduction band, which led to the formation of ROS to a large extent [99]. The singlet oxygen sensor green reagent (SOSGR) assay analysis confirmed that the ROS produced by ZnO-SQ were essentially singlet oxygen. The photodynamic antibacterial activity of the nanohybrids against *S. aureus* was confirmed by colony-forming unit (CFU) assays, which showed that the CFUs of *S. aureus* decreased by 95% after photoactivated drug treatment [99]. However, the production of ROS seems to be contradictory because some studies have revealed this mechanism in the light; other studies have reported such activities even occurred in the dark [100, 101]. The possible mechanisms of ROS generated by nano-ZnO under light and dark conditions have been discussed in a recent report [12]. Besides, Jiang et al. revealed that the addition of free radical scavengers inhibited the bactericidal effect of ZnO film against *E. coli*, suggesting that the antimicrobial ability of nano-ZnO is related to the generation of ROS [102]. The ionization of carboxyl, phosphate, and amino groups on the cell surface brought negative charges to the cell, which greatly affected the accumulation of nano-ZnO on the cell surface. Additionally, negatively charged anions, such as superoxides and hydroxyl groups, cannot enter the cytoplasmic membrane [103]. Therefore, these species exist on the outer surface of bacteria. In contrast, two HO· can be recombined to form one H_2_O_2_, which can pass through the cell wall of bacteria, subsequently disrupting the outer membrane, causing leakage of cytoplasmic contents, and DNA damage, eventually triggering cell death [65, 104]. However, excessive ROS may cause cell damage and lead to disease in the body [42, 105], which indicates the need for controlled ROS production and release as well as rational design of ZnO application solutions on the surface of Ti implants.

#### Release of Zn^2+^ ions

One of the essential mechanisms of nano-ZnO antibacterial activity is the release of Zn^2+^ ions in aqueous media. The Zn^2+^ ions are released rapidly in the first few days and then gradually reach a steady-state in the following months [54, 65, 81]. The Zn^2+^ ions released from ZnO could potentially interact with bacterial surfaces, altering charge balance, and inducing cell deformation and bacteriolysis [106]. Zn^2+^ ions cause conformational changes in the enzymes, resulting in distortion of the active sites in the enzyme as well as competitive or non-competitive reversible inhibition [12]. The reaction of Zn^2+^ ions released by nano-ZnO with the plasma membrane, as well as the cell entry of Zn^2+^ ions, can destroy the ion balance in the cells, resulting in the death of bacteria. Besides, Zn^2+^ ions can be released from dead bacteria and then act on other bacteria, leading to a long-term antibacterial process [56]. It was found that Gram-positive bacteria were not sensitive to the damage caused by Zn^2+^ ions, because the toxicity of Zn^2+^ ions embedded in negatively charged peptidoglycans was less than that of thin peptidoglycan layered Gram-negative bacteria [107]. However, some researchers reported that the inhibitory effect of the coatings containing nano-ZnO on Gram-positive bacteria was more significant than that on Gram-negative bacteria under the same conditions [108]. These contradictory results may indicate that the antibacterial activity of Zn^2+^ ions is limited. Therefore, although the dissolution of Zn^2+^ ions has been adopted and accepted as an antibacterial mechanism, it is still under discussion.

#### Other possible mechanisms

Nano-ZnO has a bactericidal effect, which depends on destroying and disordering the bacterial membrane, finally leading to the internalization of nano-ZnO into bacteria. The internalization of nano-ZnO is controlled by its particle size, surface chemistry, defects, and functionalization. Nanoparticle internalization further leads to inhibition of energy metabolism in bacteria [109]. It is reported that nano-ZnO had a positive charge in aqueous suspensions, and the Zeta potential of ZnO was + 24 mV [110], while there was a negative charge on the bacterial surface at physiological pH values. Such a reverse charge can enhance the overall effect by generating electrostatic forces, which are a secure link between nanoparticles and the surface of bacteria [109].

Due to electrostatic forces, nano-ZnO is deposited on the bacterial surface, resulting in a curtailed cell growth rate [111]. The nanoparticles interact with and accumulate on the outer surface of the plasma membrane, which increases the surface tension and induces depolarization by neutralizing the surface potential. This electrostatic interaction leads to membrane permeability and leakage of cytoplasmic fluid, which in turn leads to cell death [65, 112]. However, studies have suggested that electrostatic interactions are not the unique mechanism responsible for the antibacterial activity of nano-ZnO. The surface defects of nano-ZnO, such as corner defects, edge defects, and chemical defects, have a significant effect on the antibacterial activity that induces cell wall mechanical damage [113, 114]. In the same way, the morphology of nano-ZnO also causes physical damage to bacteria [65]. In addition, nano-ZnO plays an essential role in both immunomodulatory and antimicrobial applications. Through co-culture with innate immune cells and bacteria/lipopolysaccharide, Wang et al. found that a ZnO ‘nanoreservoir’ could enhance the phagocytosis of macrophages and inflammatory cytokine secretion of polymorphonuclear leukocytes, which contributes to its good antibacterial ability [66]. Hence, the exact mechanism of the antibacterial action of ZnO is not fully understood, and related research is required to validate the antibacterial contribution of each possible mechanism.

### Antimicrobial activity of nano-ZnO modified Ti implants

#### Antimicrobial activity of a single nano-ZnO coating on Ti implants

**Fig. 2:**
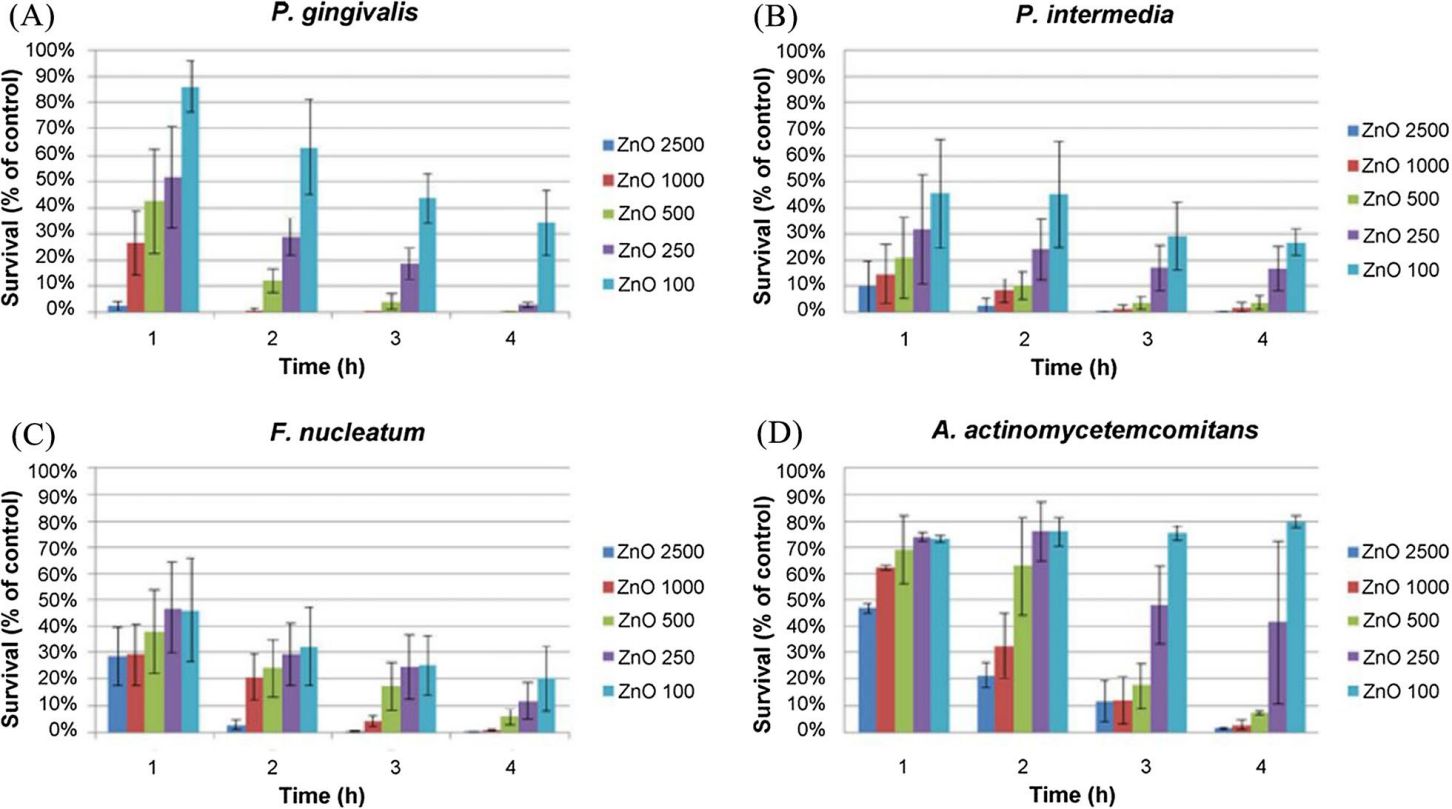
Survival rates of four anaerobic bacteria treated with five different concentrations of nano-ZnO for 1, 2, 3, and 4 h [44]

Antimicrobial studies using four types of Gram-negative bacteria associated with peri-implant periodontitis were conducted under anaerobic conditions, and the cells were stimulated with nano-ZnO at the concentrations of 2500, 1000, 500, 250, and 100 µg/mL, respectively. The results showed that each species had a significant dose-dependent effect on each species [44] (Fig. 2).

Numerous studies have verified that antibacterial ability was enhanced with increasing ZnO content [51, 57, 58]. Liu et al. confirmed that the addition of an appropriate concentration of ZnO to Ti (the initial concentration of Zn(NO_3_)_2_ was 0.015 M) could provide excellent osteogenic properties and intense antibacterial activity for stem cell differentiation of osteocytes [74]. However, Roguska et al. reached different conclusions when obtaining different amounts of ZnO nanoparticles by changing the electrodeposition time, and they observed that 3 min of loaded nano-ZnO was most effective in *S. epidermidis* killing efficacy, while more loaded nano-ZnO resulted in decreased bactericidal activity [54]. Previous studies have attributed this to a reduction in the specific surface area of modified Ti samples as well as lower solubility of aggregated nanoparticles [115]. Therefore, the content of nano-ZnO on Ti substrates should be taken into consideration. If the nano-ZnO layer is too thick, the overdose effect will have a negative influence on the antibacterial activity of nano-ZnO.

Nair et al. used ZnO particles with sizes from 1.2 μm to 40 nm, and the antibacterial activity of ZnO gradually increased accordingly [116]. The surface of the nanophase had a higher roughness than that of micro-phase ZnO. The specific surface area of the nanophase was 25% higher than that of micro-phase ZnO. Studies have shown that nano-coating may reduce the adhesion of *S. epidermidis*, thus improving the efficacy of orthopedic implants [117]. In addition, the relationship between ZnO particle size and antibacterial properties was also verified by Pang et al. [108].

**Fig. 3:**
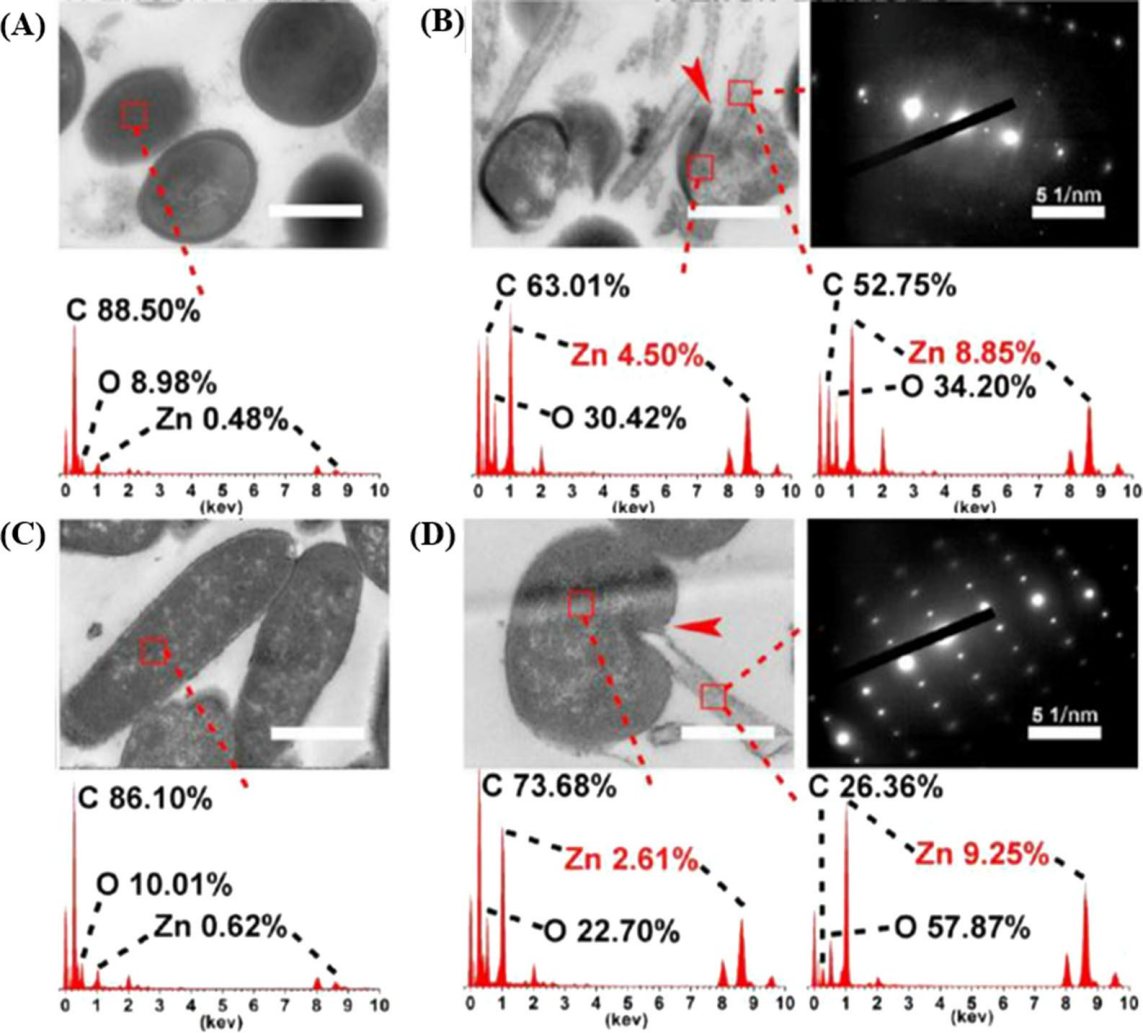
TEM images of sections and the related EDS results: **A** *S. aureus* treated with ZnO seeds, **B** *S. aureus* treated with ZnO nanorods, **C** *E. coli* treated with ZnO seeds, and **D** *E. coli* treated with ZnO nanorods (scale bars = 500 nm) [65]

Many studies have previously shown that the intensity of the antimicrobial ability of biomaterials was significantly influenced by their surface morphology, and researchers have compared the antibacterial effects of nano-ZnO with different morphologies [89, 118]. In contrast to irregular ZnO nanoparticles, ZnO nanorods on the TNTs surface can penetrate the bacterial membrane more efficiently and improve the antibacterial performance [56]. Similarly, Li et al. compared ZnO nanorod samples with ZnO seed layer samples (ZnO particles with an average size of 20 nm), and found that the tendency of Zn^2+^ ion release and the amount of ROS production was similar in both groups. Transmission electron microscope (TEM) images showed the membranes of bacteria cultured on ZnO nanorods were severely damaged, but the membranes of bacteria in another group were normal in structures and regular in shapes [65] (Fig. 3). These results revealed that the ZnO nanorod structure possessed essential contact-killing antibacterial activity.

**Fig. 4:**
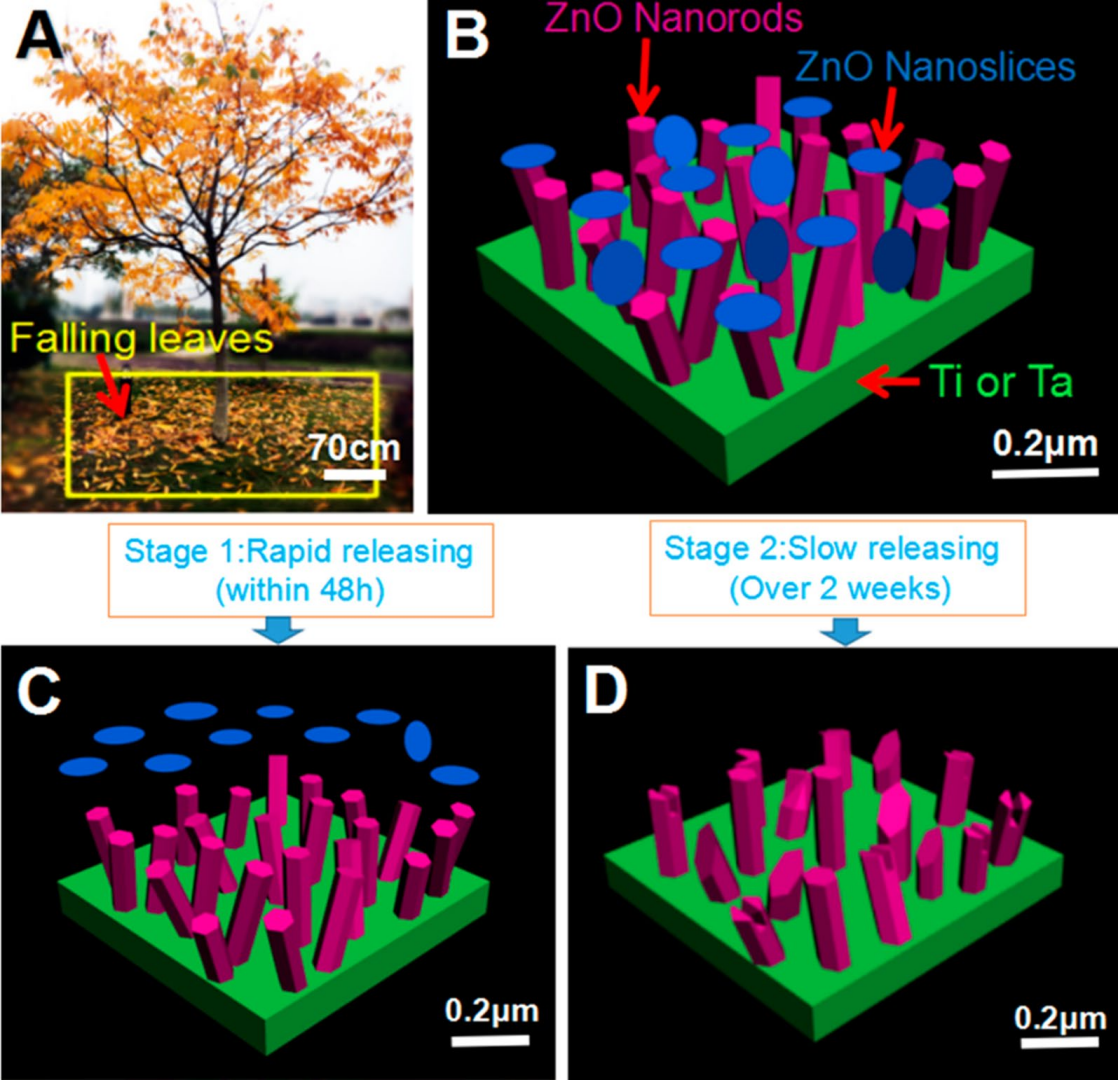
Three-dimensional schematic diagram of the bilayer ZnO-NHS: **A** conception from the trunk-leaf model **B** layered structure of trunk-like ZnO nanorods and deciduous ZnO nanoslices on the substrate surface; The two-stage release: **C** fast-release phase and **D** slow-release phase [83]

Even though the implant is more susceptible to bacterial contamination during the operation [30], the durability and stability of the antimicrobial function of the implant should not be overlooked, as it is related to the resistance to bacterial infection and the long-term success of the implant. As reported, the change of Zn^2+^ ions release concentration in modified Ti by hydrothermal growth method slowed down significantly at 12 days [65], while similar results were achieved in 4 days using laser cladding technology [46]. This phenomenon indicated that different surface modification techniques resulted in variable stability of nano-ZnO modified Ti. Further studies demonstrated that PLGA coatings introduced to the surface of ZnO nanorods fabricated by hydrothermal growth technique continue to prolong the release of Zn^2+^ ions [76]. It implied that the long-lasting antimicrobial effect could be controlled by the modification technique approach and the introduction of polymer coatings, but the explanation for the variations of the different modification methods has not so far been reported and subsequent studies could be discussed in terms of the bond strength of nano-ZnO to the substrate and potential variance of surface morphology in dissolution rates. The single morphological structure of ZnO nanoarrays was insufficient to provide the phasic function in bacterial inhibition. Inspired by fallen leaves, Liao et al. constructed a hierarchical structure in ZnO nanorods-nanoslices (NHS) to modify the implant surface, which achieved the rapid release of Zn^2+^ ions in the early stage (within 48 h) and immediately killed the bacteria around the implant. In the second stage (more than 2 weeks), the NHS showed slow release to achieve long-term inhibition. The good antibacterial activity of ZnO-NHS was confirmed in animal experiments in vivo [83] (Fig. 4). Subsequently, a bilayer biomimetic nano-ZnO for dental implants constituting nanorods and nanospheres was found to have similar antibacterial effects, which was confirmed by exposure to the same *S. aureus *and* E. coli* strains [79]. Until now, almost all of the present studies have shown antibacterial effects against aerobic bacteria, but few studies have examined anaerobic bacteria.

As well, more attention should be given to the method of nano-ZnO use, ensuring its interaction with bones or tissue cells and that the implant shows no deleterious effects. Nano-ZnO coatings tend to increase the cytotoxicity of implants while improving their antibacterial capacity. Therefore, it is important to strike a balance between antibacterial capability and biocompatibility [119, 120]. For example, Zhang et al. used the hydrothermal treatment to incorporate ZnO into MAO-TiO_2_ coatings to generate a hierarchical micro/nanostructure, which has vigorous antibacterial activity but has certain toxicity toward osteoblast-like cells. However, the simple heating treatment improved cytocompatibility by increasing the crystallinity of the coating, resulting in a slow and stable release of Zn^2+^ ions [47]. Li et al. designed hybrid ZnO/PDA/arginine-glycine-aspartic acid-cysteine (RGDC) nanorod arrays on Ti implants. It was also shown that the coverage of PDA and RGDC could simultaneously inhibit ROS accumulation and increase Zn^2+^ ion concentrations, thereby improving cell compatibility [65].

**Fig. 5:**
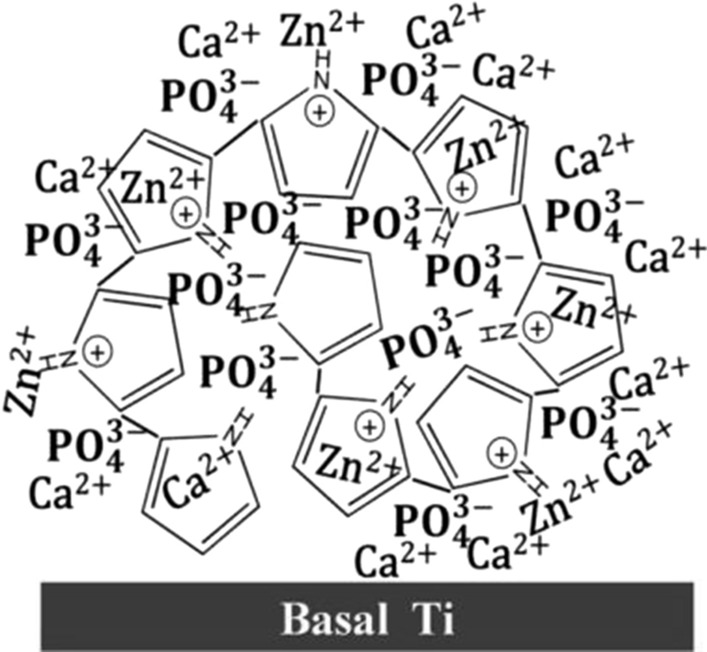
Schematic illustration of PPy as a dual regular of nHA and nano-ZnO [61]

HA has almost the same chemical composition as natural bone and teeth, and the size and rigidity of nano-sized HA (nHA) are highly similar to those of HA crystals in natural bone; thus HA has good biocompatibility and osteoinductive activity, and is often used as a coating material [121, 122]. Therefore, some scholars obtained better biocompatibility and osteoinductivity while achieving a good antibacterial effect when nano-ZnO was doped with nHA [70, 75]. In the latest research, polypyrrole (PPy) was used as a dual regulator of nHA and nano-ZnO through coordinating and doping of ions, which reduced the release rate of Ca^2+^ and Zn^2+^, not only ensuring a good antibacterial effect but also improving the physiological stability of the composite coating [61] (Fig. 5).

In vivo implant infections involve interactions among the implant, the bacteria, and the local host immune response [123]. Mononuclear macrophages and polymorphonuclear leukocytes (PMNs) are two essential cells in the natural immune response and serve a pivotal function in the innate defense of the host against bacterial infection [124]. To investigate the potential effect of nano-ZnO films on Ti surfaces in natural immunity against bacterial infection, Wang et al. co-cultured murine RAW 264.7 macrophages and PMNs with bacteria and found that bacterial phagocytosis was greater in all the nano-ZnO groups than in the Ti group [66]. To further understand the reason for this, ZnO films were co-incubated with macrophages exposed to bacterial irritation, and the results showed that the release of pro-inflammatory cytokines, i.e. TNF-α, IL-6, monocyte chemoattractant protein-1 (MCP-1), was increased in the nano-ZnO group, and thus the overall effect of nano-ZnO films promoted the local inflammatory response, resulting in the recruitment of more immune cells to fight bacterial infections. Interestingly, the secretion of anti-inflammatory cytokines (IL-10), which are antagonists of pro-inflammatory cytokines and prevent uncontrolled inflammatory responses [125], was also increased. However, in another report [56], nano-ZnO was used to enhance the biological properties of Ti-based implants by inhibiting macrophage activity and weakening the inflammatory response while exerting its antimicrobial efficacy. Although the current research remains controversial, it is obvious that nano-ZnO could modulate the immune response to assist in the elimination of bacterial infections, and future works should focus on the mechanism of action of nano-ZnO on inflammatory factors to control the inflammatory response.

#### Antibacterial activity of nano-ZnO and other materials on Ti implants

**Fig. 6:**
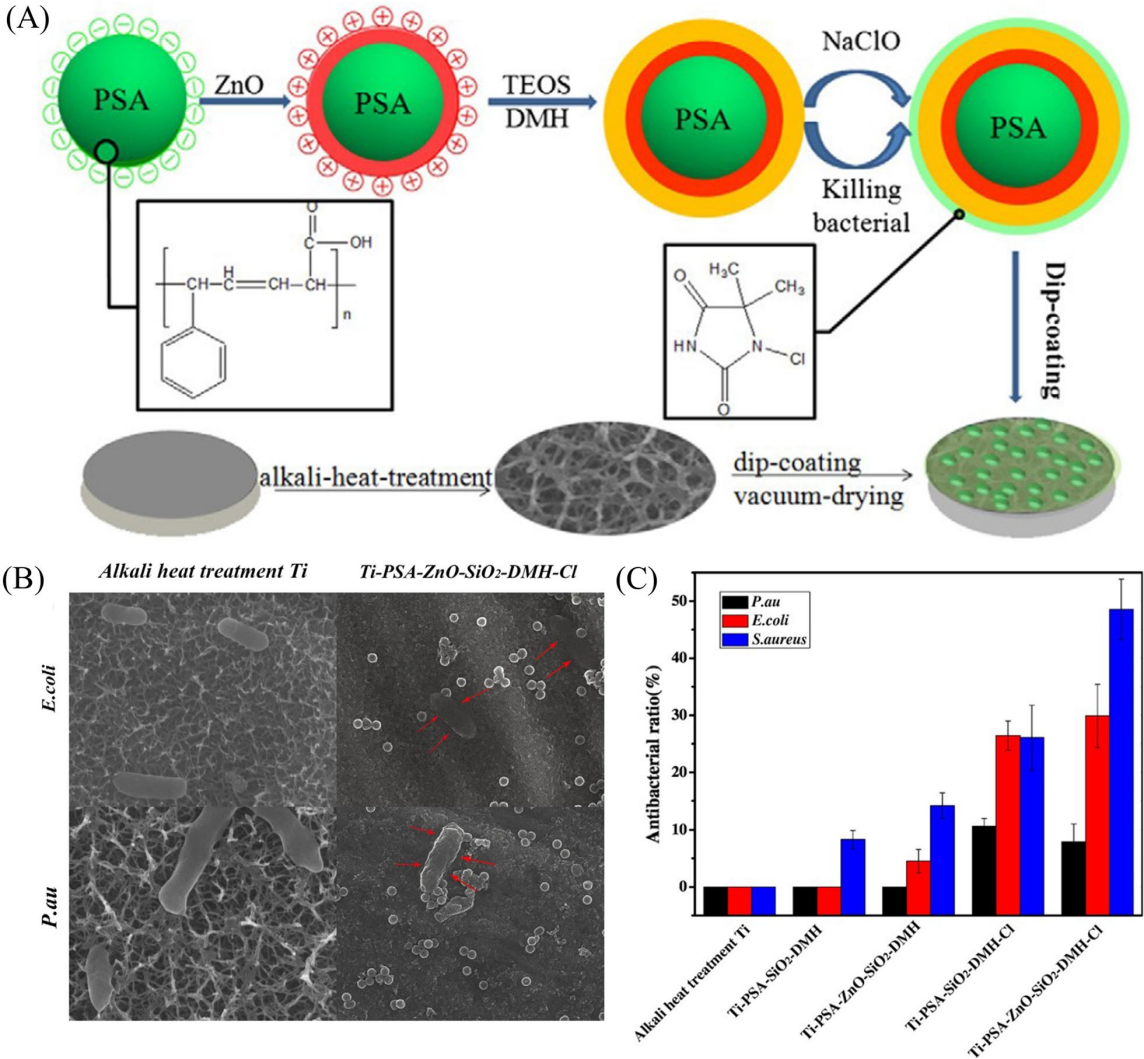
**A** The preparation process for Ti-PSA-ZnO-SiO_2_-DMH-Cl composite material. **B** Scanning electron microscopy (SEM) patterns of *P. aeruginosa* and *E. coli* on samples (the scale bar is 1 μm). **C** Antibacterial activity of a hybrid system composed of N-halamine and ZnO nanoparticles against *P. aeruginosa*, *E. coli*, and *S. aureus* [78]

Using different composites can reduce the cost of potential applications and the possibility of bacterial drug resistance by optimizing the concentration of individual nanoparticles [44]. To further improve the bactericidal characteristics, the combination of nano-ZnO and other antimicrobial agents has been extensively studied. Recently, surface functionalization with organic antimicrobial agents has also been considered to obtain antibacterial properties on Ti surfaces [126, 127]. Antibacterial N-halamine polymers and related coatings have been widely studied in the past decade because of their strong broad-spectrum antibacterial activity against microorganisms, long-term chemical stability, renewability, biosafety, environmental friendliness and low cost [78]. Li et al. built hybrid nanoparticles that were immobilized on a Ti surface via hydrogen bonding, endowing Ti implant material with potent antibacterial ability. And the hybrid nanoparticles were composed of N-halamine and nano-ZnO on the surface of colloidal templates [monodispersed polystyrene-acrylic acid (PSA)]. To investigate the antibacterial activity of the nanoparticles, the samples were incubated with *P. aeruginosa*, *E. coli* and *S. aureus*. The results showed that ZnO or DMH-Cl (DMH, the precursor of N-halamine) has an obvious antibacterial effect, and the combination of ZnO and DMH-Cl could enhance antibacterial efficiency [78] (Fig. 6).

**Fig. 7:**
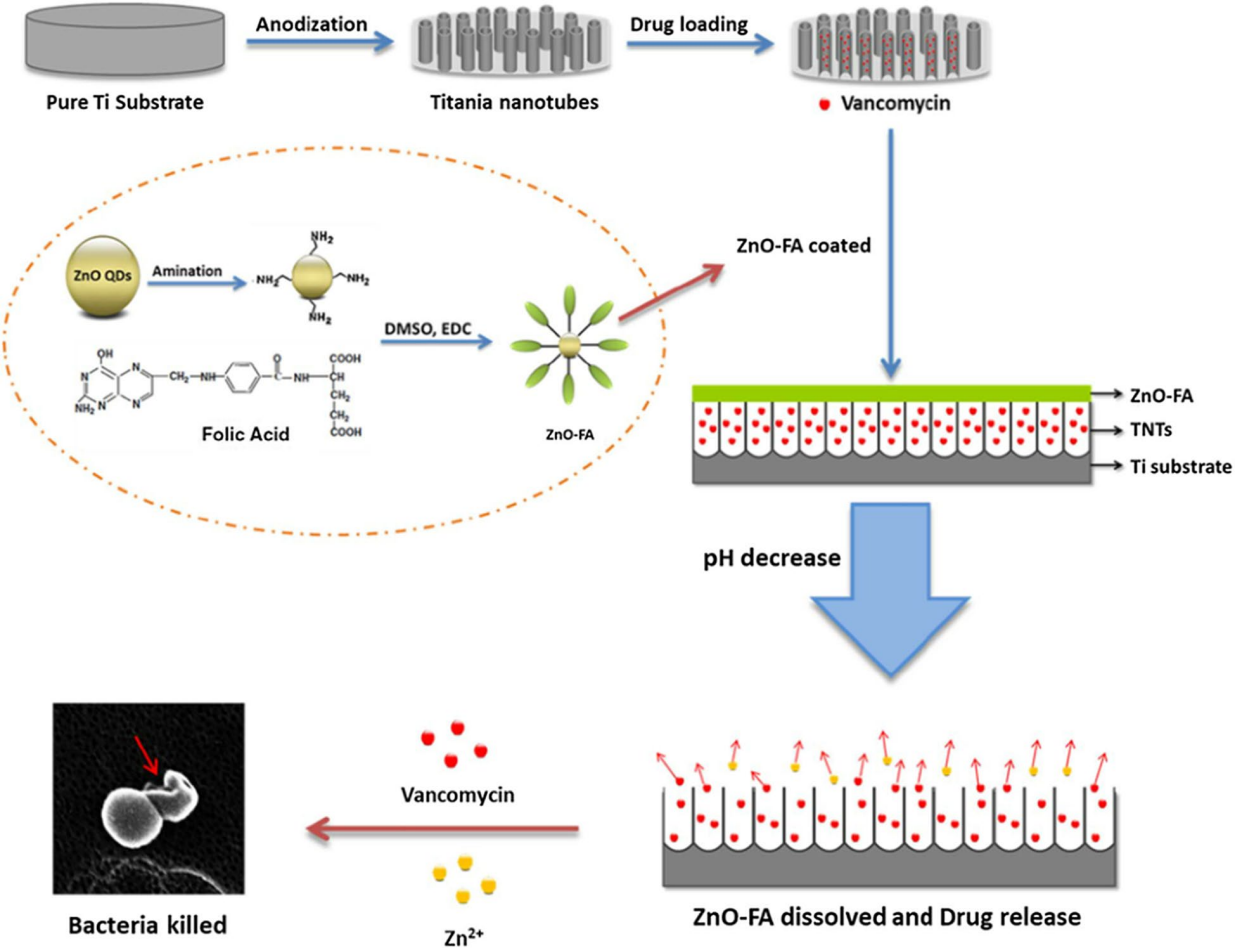
Schematic diagram of preparation process of TNTs-Van@ZnO-FA system and the mechanism of pH-triggered synergistic sterilization [81]

In clinical practice, antibiotics are usually prescribed to prevent infection-related complications [7]. However, there are still many doubts and conflicting opinions on several aspects of using antibiotic implants after years of scientific debate, because of mutant resistance produced by long-term exposure to antibiotics below the inhibitory concentration, serious side effects, and other factors [128]. Ti implants topically loaded with antibiotics can provide sustained drug release, but non-specific release and local overdose issues make them detrimental to long-term antimicrobial action [129]. Designs with condition-response mechanisms are likely to be considered as a more refined solvent [130, 131]. Xiang et al. fabricated a pH-sensitive ZnO-FA-blocked TNT drug delivery system [81]. Using TNTs modified with the antimicrobial drug vancomycin (VAN) as a drug delivery platform, folate (FA) was coupled on the surface of ZnO quantum dots (QDs)-NH_2_ by an amidation reaction to construct the blocking layer. Because of the protective effect of ZnO-FA on the surface of TNTs, the system was stable under physiological pH, while the dissolution of ZnO-FA in an acidic environment was caused by bacterial infection, thus releasing VAN, and the antibacterial ratio in the TNTs-VAN-ZnO-FA groups increased significantly with the decrease of pH (from 60.8 to 98.8%). Overall, the results showed that the drug delivery system has potential application prospects in bacteriostasis (Fig. 7).

**Fig. 8:**
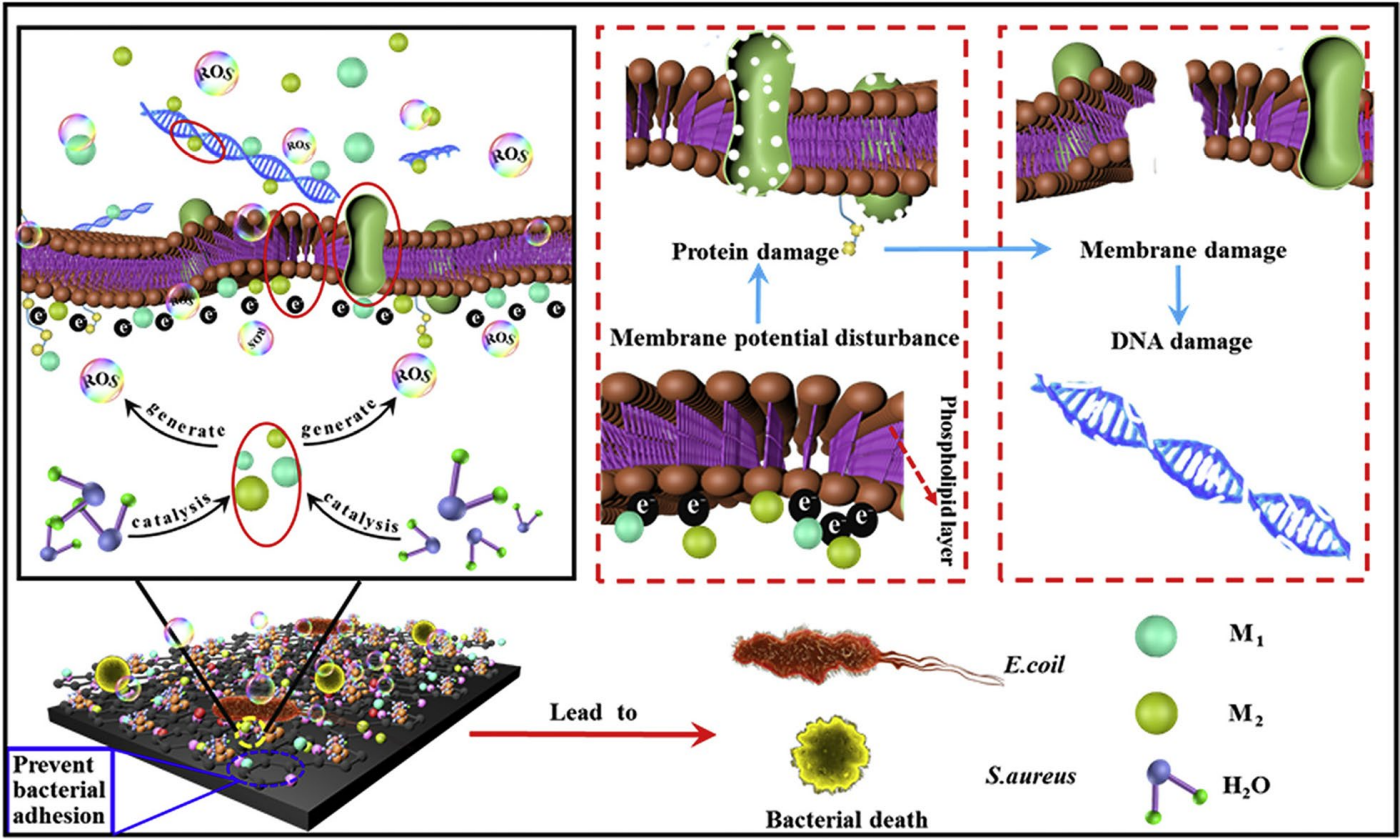
Mechanism diagram of multi-metal nanoparticles composite coating against *E. coli* and *S. aureus* [91]

Some scholars have found that the antibacterial efficacy of nano-ZnO against Gram-positive bacteria such as *S. aureus* is better than that against Gram-negative bacteria such as *E. coli*. Complementary to nano-ZnO, nano-Ag shows potent antibacterial efficacy toward Gram-negative bacteria. In combination, Ag/ZnO nanohybrid materials exhibit more obvious antibacterial efficacy [81]. Shang et al. prepared nano-Ag modified ZnO nanorod arrays and tested them against *P. aeruginosa*. Compared with other samples, the morphological structure of ZnO nanorods optimized the size and distribution of nano-Ag, endowing the nanorods with excellent synergistic antibacterial properties [77]. The synergistic effect of nano-ZnO and nano-Ag greatly improves antimicrobial efficiency and reduces the cytotoxicity of high doses of nano-Ag and the cost of antimicrobial coatings. As mentioned above, the antibacterial function of nano-ZnO is highly dependent on ion-releasing behavior; excessive release of Zn^2+^ can produce cytotoxicity [132]. Xiang et al. solved this problem by using a sol-gel method to prepare PLGA/Ag coatings on a Ti disk with ZnO nanorods [76]. By using PLGA overlays to encapsulate two powerful inorganic antibacterial agents, ZnO and nano-Ag, the Ti implants exhibited superior antibacterial ability while avoiding potential cytotoxicity effectively [76]. Further results showed that cellular responses varied with different initial concentrations ratios of Ag/ZnO, indicating that the nano-Ag/ZnO-embedded HA coating(Ag/ZnO/HA=7: 3: 90 wt%) exhibited the best antibacterial effect and osteogenic ability, exemplified by the broad-spectrum antibacterial efficacy of 96.5 and 85.8% against *E. coli* and *S. aureus*, as well as by the enhanced osteoinductive ability [46]. Lately, a uniform PPy-HA/ZnO-Ag-Cu nanocomposite coating was constructed on Ti substrates using PPy as a regulator of HA nanoparticles and metal particles (Fig. 8). In comparison with the pure HA coating, the multi-metal nanoparticles doped coating not only exhibited 100% inhibition of both *S. aureus* and *E. coli*, but also presented the required bioactivity, namely, cytocompatibility, angiogenic, and osteogenic capability as a promising candidate coating material for Ti implants [91].

Chitosan (CS) is an antimicrobial polymer with excellent biocompatibility [133]. As a notable strategy, ZnO-containing CS-based composite coatings and their derivatives demonstrate notable potential for antibacterial applications based on antibiotic-free strategies, including cationic contact killing surface structures and the inherent antibacterial characteristics of nano-ZnO. It was detected that the CS/nano-ZnO coating showed 1.2-fold stronger antibacterial activity against *E. coli* than the CS coating alone and actively prevented the formation of biofilm [80]. Further study was carried out by Lin et al., where a double-layer antimicrobial coating design on pure Ti was proposed [59]. The new double-layer included an inner layer of TNTs doped with nano-Ag (TNTs/Ag) and the outer layer of CS-gelatin (Gel) mixture containing nano-Ag and nano-ZnO (CS-Gel-Ag-ZnO). Further studies on the antimicrobial activities of the coating against planktonic and adherent bacteria revealed that the limited inhibitory effect of nano-Ag against planktonic bacteria in TNTs was compensated by the CS-Gel-Ag-ZnO layer. The antimicrobial capability of the novel composite coating of TNTs/Ag+CS-Gel-Ag-ZnO was enhanced due to the intrinsic synergistic antimicrobial activity of CS, nano-ZnO, and Ag against planktonic bacteria. The extremely high antibacterial rate of 99.2% shows a broad prospect in the field of orthopedic surgery and dental implants [59].

The hydrophilicity/hydrophobicity of a material surface is also an important factor affecting bacterial adhesion. Zhang et al. obtained a superhydrophobic Ti substrate by grafting octadecylphosphonic acid (OPDA)-toluene to resist bacterial adherence. Furthermore, OPDA modification adjusted the release rate of Zn^2+^ ions to achieve long-term release and consequently improve the biocompatibility of Ti-based metallic implants in regard to antibacterial effects [64].

## Osteogenic induction of Ti surface containing nano-ZnO

Zinc is a trace element in the human body that is essential for the growth of the skeleton [134]. In previous studies, nano-ZnO was incorporated into the biomaterial surface to promote implant osseointegration and shorten the bone healing period. Here, the major form induced by osteogenesis of nanoZnO-based Ti-implants is summarized.

### Effect of nano-ZnO modified Ti implants on osteoblasts

**Fig. 9:**
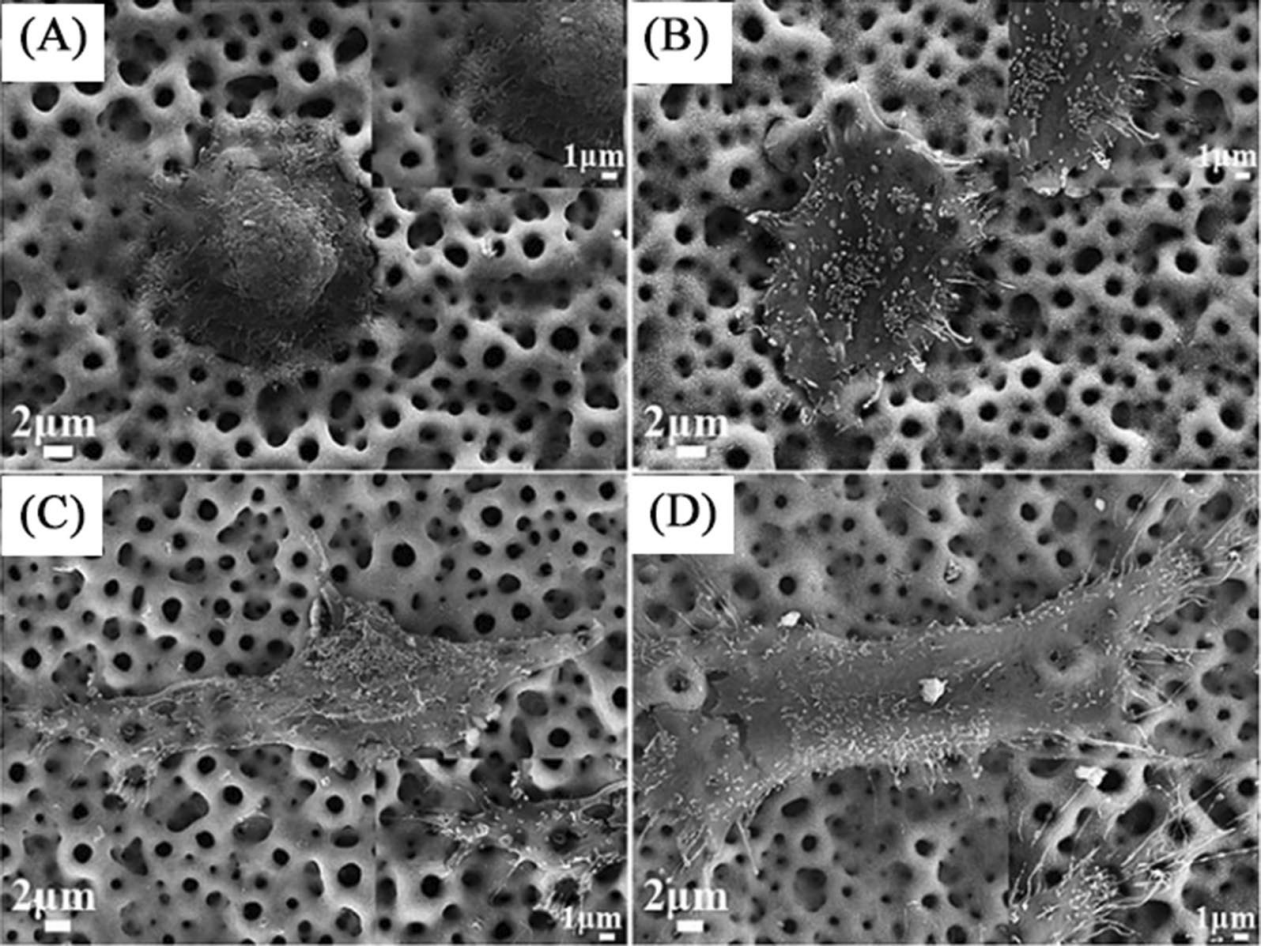
SEM images of morphologies of SaOS-2 cells adhered to the MAO: **A** 4 h and **C** 24 h; MHTZn: **B** 4 h and **D** 24 h [136]

The physicochemical features at the interface of biomaterials contribute significantly to the regulation of cellular responses [41], and several studies in recent years have reported the effects produced by various nano-ZnO modified Ti surfaces on cells. Studies have reported that a well-defined and controlled Zn-incorporated nanotopography on the acid-etched pure Ti surface by a hydrothermal treatment was generated, and the results showed that the modification of the acid-etched Ti with nanostructured ZnO enhanced the proliferation and alkaline phosphatase (ALP) activity of osteoblast-like SaOS-2 cells in a dose-dependent manner [135]. In a subsequent study, a novel micro/nanostructured TiO_2_/ZnO coating (MHTZn), produced by MAO, hydrothermal treatment, and heat treatment, was designed to achieve a balance between antibacterial activity and cytocompatibility. An increased number of filopodia were observed under SEM compared to the MAO group (Fig. 9). In vitro biological experiments demonstrated that the micro/nanostructured coating with bioactive Zn^2+^ ions had significantly promoted cell adhesion and the expression of osteogenic activity markers, including ALP activity, collagen secretion, osteopontin (OPN), and osteocalcin (OCN) in SaOS-2 cells [136]. Similarly, in another investigation, in vitro experiments with commercially available human osteoblasts showed increased osteoblast adhesion, ALP activity, and calcium mineral deposition on nanostructured ZnO and TiO_2_ compared to microphase formulations. In addition to Zn^2+^ ions, the surface morphology of the coating forms local microenvironments for osteoblast growth and regulates the behavior of bone-related cells [117]. However, the excessive Zn^2+^ ion concentration inhibited cell growth. It has been reported that osteogenic activity is enhanced by the introduction of a biodegradable PLGA coating that regulates the release of Zn^2+^ ions [76]. Hence, more emphasis should be given to the correlation between the physicochemical properties of ZnO and the osteogenic activity of osteoblasts.

### Effect of nano-ZnO modified Ti implants on osteoclasts

Osteoclasts are a cell type involved in the process of osseointegration, as opposed to osteoblasts [21]. Abnormally high osteoclast activity could result in excessive bone resorption. Consequently, reducing excessive osteoclastic-induced bone resorption would facilitate implant-bone integration [16]. It was reported that the binding of RANKL to RANK activates nuclear factor κB (NF-κB), which then induces osteoclastic differentiation [137]. Zn suppressed osteoclast differentiation by antagonizing the activation of (NF-κB) and RANK expression as well as the activation of extracellular signal-regulated kinase (ERK) [138, 139]. But, controversial results of osteoblasts being differentially subject to nano-ZnO were observed. In bone metabolism, tumor necrosis factor (TNF)-α is considered a key cytokine in inflammatory osteoclastogenesis and bone destruction. Choi et al. applied nano-ZnO to cause bone resorption in mouse calvarial bone in vivo, and hypothesized that it possibly relates to Zn^2+^ ions-induced TNF-α release to activate osteoclasts [140]. The interaction between osteoclasts and nano-ZnO is quite complex, with no consistent results from current studies and few studies based on nano-ZnO modified Ti surfaces. Future implant materials should not only inhibit osteoclast-mediated bone resorption, but also modulate a dynamic balance among osteoblasts-materials-osteoclasts to achieve stable osteointegration over time.

### Effect of nano-ZnO modified Ti implants on the osteogenic differentiation of mesenchymal stem cells (MSCs)

It is widely known that MSCs can be induced to differentiate into osteoblasts under specific conditions at the biomaterial interface [141]. Oh et al. found that intense stem cell elongation induced cytoskeletal stress and selective differentiation into osteoblasts to adapt to the force [142]. Subsequently, a clear research result demonstrated that the bone mesenchymal stem cells (BMSCs) on Zn-incorporated TiO_2_ coatings were subjected to abnormal tension and stress. According to previous research, Zn-incorporated TiO_2_ coatings had an obvious biological activity to promote the differentiation of rat BMSCs [51]. Further study suggested that the nano-ZnO was doped into TNTs by Liu et al., with the aim of optimizing the differentiation of MSCs and antimicrobial properties of the original Ti substrate. ZnO nanoparticles with tunable concentrations were incorporated into TNTs by Liu et al. using a facile hydrothermal strategy, with the aim of optimizing mesenchymal stem cell differentiation and the antibacterial properties of Ti. Through response surface mathematical model simulation and experimental verification, Ti incorporated with ZnO at appropriate concentrations (with an initial concentration of Zn^2+^ ions at 0.015 M) can provide exceptional osteogenic properties for the differentiation of MSCs among bone cells with strong antibacterial effects. Compared to the control groups, ALP activity improved to approximately 13.8 U/g protein, and the osteocalcin, collagen-I, and osterix gene expression in mesenchymal stem cells also improved [74].

### Effect of nano-ZnO modified Ti implants on immune cells

Existing osteogenic mechanisms are mainly described in terms of osteoblast differentiation, largely ignoring the influence of immunomodulation on osteogenic activity. The implant is considered a foreign body during the osseointegration process [143]. The host develops immune responses after implantation, and macrophages are among the first cells to interact with implants, which occupy a vital position in the innate immune defense [144]. As the precursor of osteoclasts, macrophages can release a variety of proteins, factors, and cytokines involved in both immune regulation and bone regulation [145, 146]. These secreted factors further affect extracellular matrix deposition and new bone formation [147].

Culturing macrophages on a nanoZnO-modified Ti implant (TNTs/ZnO) revealed that TNTs/ZnO has a significant inhibitory effect on the proliferation and adhesion of macrophages and could be used to prevent chronic inflammation and control the inflammatory reaction [56, 148]. Moreover, the inflammatory responses of macrophages on the micro/nanostructured TiO_2_/ZnO coating were investigated by Zhang et al. [53], and these authors showed an increase in the expression of IL-4, IL-6, IL-10, and TNF-a in the MHTZn (the micro/nanostructured TiO_2_/ZnO coating) group compared to the MAO (macroporous TiO_2_ coating) group. When polarized into the M1 phenotype, macrophages mainly secrete pro-inflammatory cytokines such as TNF-α, IL-6, and IL-12, whereas, in the M2 phenotype, the main secreted cytokines are anti-inflammatory cytokines such as IL-4, IL-10, and TGF-β [144]. In this study, the increase in pro- or anti-inflammatory cytokines indicated that macrophages were polarized toward the M1 and M2 phenotypes, respectively. To further verify these results, they collected the medium of macrophage cell line RAW264.7 cultured on the surface of MAO and MHTZn substrates and used it as the conditioned medium to culture SaOS-2 cells. The results showed that no significant differences in proliferation were observed between the cells cultured in these two conditioned media, whereas SaOS-2 cells cultured in MHTZn conditioned medium showed an upregulation of ALP activity and increased mineralization. These findings not only confirm that MTHZn promotes the polarization of macrophages, but also simultaneously indicate that nano-ZnO modified Ti surfaces can enhance osteogenic properties by affecting immune cells [53]. Although the influence of macrophage cells on nano-ZnO has been confirmed, other immune cells, such as B cells and T cells, remain to be explored.

### Effect of the piezoelectricity of nano-ZnO modified Ti implants on bone formation

It is well known that electrical stimulation affects the process of bone regeneration by changing the cellular response. Because of the ability to generate charge/potential in response to mechanical deformation, nano-ZnO piezoelectric materials exhibit promising potential in the fabrication of smart stimulation scaffolds for bone regeneration [149].

Pang et al. used a Bose dynamic biomechanical reactor to periodically load on the surface of the ZnO/TiO_2_ coating to investigate its effects on the spreading, proliferation, and differentiation of MC3T3-E1 osteoblasts. After applying periodic loading, the cell proliferation rate and the number of cell pseudopods on the coating material were increased. However, loading on the surface of Ti without piezoelectric effect had no significant effect on cell proliferation and differentiation compared with that without loading. Moreover, after loading, the expression of ALP on Ti surface coatings with different particle sizes of nZnO/TiO_2_ (Z1 < Z2 < Z3) increased significantly compared with unloaded coatings; the expression of ALP on Z1, Z2, and Z3 coatings was significantly higher than that of unloaded Z1, Z2, and Z3 coatings. These results confirmed that the significant improvement in the cytocompatibility and osteogenic ability of MC3T3-E1 could be attributed to the piezoelectric properties of ZnO/TiO_2_ under the periodic loading. Besides, because the Z1 coating had the largest piezoelectric coefficient and produced the most charge, the highest expression of ALP was obtained. It showed the particle size of ZnO/TiO_2_ is negatively correlated with the piezoelectric properties, cytocompatibility, and osteogenic properties of the composite coatings. Although ZnO/TiO_2_ piezoelectric composites show good piezoelectric properties under periodic loading and can promote the activity of osteoblasts in vitro, in vivo testing of this smart biomaterial remains to be verified [108].

### Effect of nano-ZnO modified Ti implants on neovascularization formation

It is well-known that the formation of neovascularization is extremely valuable for bone regeneration, as it promotes nutrient delivery and bone repair [150]. Thus, clinical success depends not only on the osseointegration around the Ti implant, but also on the neovascularization [16]. Various growth factors secreted by osteoblasts, e.g. vascular endothelial growth factor (VEGF), fibroblast growth factor-2 (FGF-2), and angiopoietin 1, are regulated by the morphology and energy of the Ti implant surface [151, 152]. It was reported that Zn is an important component of early growth response 3 (Egr3) which has an essential downstream role in VEGF-mediated endothelial functions leading to angiogenesis [153]. Maimaiti et al. cultured VECs on the surface of the composite coating and observed that the adherence and spreading of VECs improved with the addition of nano-ZnO within the composite coating [61].

## Anti-corrosion property of nano-ZnO modified Ti implants

Metals in implant devices are prone to corrosion, thereby disturbing the homeostasis of osteoblasts [154]. Corrosion of the metal layer of the implant may be due to the cracked and porous nature of the coating, which makes it permeable. Electrolytes penetrate these pores and come into contact with the substrate surface, thus initiating the corrosion process. Therefore, much research has been focused on depositing functional coatings on Ti substrates to improve the corrosion resistance in a simulated in vivo environment. It was frequently reported that the corrosion performance of Ti substrate materials decorated with nano-ZnO was significantly improved. Aydin et al. deposited nano-ZnO and nano-Ag on the surface of TiO_2_-nanotubes using hydrothermal and chemical reduction methods respectively, and found that the ZnO-TiO_2_-nanotubes had a higher resistance value, i.e., higher corrosion resistance. This result was attributed to the advantage of the ZnO nanorods structure in blocking the TiO_2_ tube channel [155]. In a study, a ZnO-doped tantalum oxide (Ta_x_O_y_) multilayer composite coating was deposited by magnetron sputtering at room temperature. The potentiodynamic polarization curves revealed that ZnO-doped multilayer composite coating had higher corrosion potential and lower corrosion current density (1.12 ± 0.004 µA/cm^2^) than that of Ta_x_O_y_ coating, showing better corrosion inhibition [67]. Roknian et al. indicated that the corrosion current density had a 93% reduction for the sample containing ZnO nanoparticles at a concentration of 15 g/L in comparison with the uncoated Ti substrate [58]. Similarly, the TiO_2_ coatings incorporated with 30 wt% nano-ZnO revealed the highest corrosion resistance and antibacterial activity due to the synergistic actions of both TiO_2_ and ZnO [156]. Besides, Vijayalakshmi et al. also made a composite coating with TiO_2_ and ZnO via an electrophoretically deposited method to enhance the activity against electrochemical corrosion. It was found that good corrosion resistance capacity was achieved at a voltage of 40 V sintered in a vacuum atmosphere compared to other coating conditions, which was attributed to a denser surface and less porosity [62]. Moreover, incorporation of Sr^2+^ and ZnO into HA significantly reduced porosity, and the SrHA/ZnO coating became significantly denser, which exhibits higher corrosion resistance in simulated body fluid than the pure HA coating [60]. Although it has been confirmed that nano-ZnO coatings could alter the corrosion performance of the original Ti substrate, differences in surface morphology with a specific chemical composition may still result in variable biological properties. Recently, it was reported that the nano-ZnO coating with spherical morphology provided high initial corrosion performance, while the irregular nanosheet with porous networks and flower-like morphology impaired the corrosion performance of the Ti6Al4V substrate [92].

To enhance the corrosion resistance of the nano-ZnO coating on a Ti substrate, nano-ZnO was functionalized with different bifunctional organic molecules. Electrochemical results indicated that the modification of Ti with nano-ZnO and organic molecules shifted the open circuit potentials to a higher level, decreased the corrosion current density, enhanced the resistance of the material, and significantly improved the corrosion resistance of pristine Ti. In particular, the APPA bifunctional molecule modified with ZnO, showed high surface energy, noble open-circuit potential (− 0.2 V) and significantly lower corrosion current density (5.3 × 10^−7^ A/cm^2^), indicating promising interactions with bioactive molecules in the biological environment and improved corrosion resistance [71], which was confirmed in another study [72]. In summary, factors such as preparation parameters and composition ratios may have an impact on corrosion resistance and remain to be further explored. In fact, since the body environment is extremely complex and variable, electrochemical experiments only indicate a trend in corrosion resistance of Ti implants, while the actual period of effective corrosion resistance in clinical use has to be assessed by extensive animal studies in vivo.

## Conclusion and perspectives

Ti is widely used in human implants due to its good biocompatibility and suitable mechanical strength, but its surface oxidation susceptibility leads to biological inertia that limits its practical use. To solve this issue, one of the hot research topics in recent years is the surface modification of implants to obtain the desired properties. Among various materials, nano-ZnO is considered to be a broad-spectrum inorganic antimicrobial agent with a good bactericidal effect against almost all known bacteria, and zinc is one of the essential trace elements in the human body. This review focused on methods for nano-ZnO modification of the surface of Ti substrates, possible antimicrobial mechanisms of nano-ZnO, as well as antimicrobial applications of nano-ZnO and Ti substrate surfaces with two or more composites containing nano-ZnO. In addition, the application of nano-ZnO in osteogenesis induction was discussed. Finally, a summary of the enhanced anti-corrosion properties of nano-ZnO was presented. The purpose of this review was to provide a reliable reference for scientists and clinicians interested in the functional applications of ZnO-nanomaterials on Ti surfaces by summarizing and discussing their applications in recent years.

Surface modification is a useful strategy for enhancing the biological properties of the Ti substrate. Now, it is considered as a central concept to develop modified Ti materials that are clinic-driven based on the characteristics of the patient’s course of the process. Nano-ZnO modified Ti-based materials have presented satisfactory (bio)physicochemical performances in current studies relevant to resistance to bacterial infection, while the cytotoxic phenomenon exhibited in some studies will necessitate a focus on balancing antimicrobial and biocompatibility in future research. Possible research hotspots involve exploring the minimum amount of nano-ZnO to inhibit bacteria on specific platform surfaces, developing smart release modes triggered upon infection, improving or exploring novel hybrid coatings and their potential antimicrobial mechanisms to reduce reliance on the original mechanisms. With the clinical requirements for slow corrosion rates and prolonged stability of the implant, changes in the integrity of the nano-ZnO-modified coating during in vivo degradation are modeled by in vitro experiments with appropriate simulated body fluid, and subsequent long-term experiments in vivo to verify and assess the osseointegration stability. In fact, nano-ZnO is comparatively rare in the literature for modified Ti compared to other conventional materials (Ag, HA, organic materials, etc.) and even scarcer for in vivo studies. More importantly, the implant material is considered a foreign invader and the body’s immune system plays an extremely important role in fighting it. In this context, the available scientific literatures have not been discussed from a comprehensive perspective. Therefore, it is necessary to construct sophisticated microenvironmental models of the human body to explore in-depth the biological changes and the underlying mechanisms that occur with various types of cells and tissues after implantation under immune response. These approaches will facilitate the design of a multifunctional nanomaterial platform for superior bio-implant materials that are efficiently resistant to bacterial infection and provide improved osseointegration efficiency, while maintaining biocompatibility or low cytotoxicity.

## Data Availability

Without restrictions.
